# Out-of-Pocket Spending for Cancer Medication, Financial Burden, and Cost Communication with Oncologists in the Last Six Months of Life in Israel

**DOI:** 10.3390/healthcare9091120

**Published:** 2021-08-30

**Authors:** Aviad Tur-Sinai, Netta Bentur, Damien Urban

**Affiliations:** 1 Department of Health Systems Management, The Max Stern Yezreel Valley College, Yezreel Valley 1930600, Israel; 2 School of Nursing, University of Rochester Medical Center, Rochester, NY 14642-8404, USA; 3 Sackler Faculty of Medicine, The Stanley Steyer School of Health Professions, Tel-Aviv University, Tel Aviv-Yafo 6997801, Israel; nettabentur51@gmail.com; 4 Sheba Medical Center, Department of Oncology, Tel Hashomer, Ramat Gan 5257354, Israel; Damien.Urban@sheba.health.gov.il

**Keywords:** cancer, out-of-pocket, cost, medication, payment, financial burden, communication with oncologists

## Abstract

Honest communication between oncologists and patients is important in alleviating the financial burden of cancer care. This study explored patient–relative–oncologist communication regarding the affordability of out-of-pocket (OOP) medication and the extent to which this communication addresses itself to the families’ financial burden. A cross-sectional survey was conducted among primary caregivers of deceased cancer patients. About 43% of relatives said that they and/or the patients had paid out of pocket for medications during the last six months of the patient’s life. Most (73%) oncologists suggested an OOP medication without asking about financial ability, 43% hardly explained the advantages of an OOP medication, and 52% hardly explained any treatment alternatives. Older age and female gender were related to less communication about an OOP medication, and better education, greater affluence, and having private health insurance were related to more communication. About 56% of relatives said that OOP payment for medications inflicted a very heavy or heavy financial burden on patients and their households. Physicians’ interest in financial ability and giving explanation lightened the burden. Given the difficulty of explaining the complex interactions of cost and clinical outcomes, oncologists need to be better educated in skills that would enable them to communicate costs more openly and should consider the cost of a treatment when prescribing it.

## 1. Introduction

Cancer is one of the most expensive medical conditions in the United States [1,2,3,4]; in Europe, too, its cost is rising considerably and more quickly than in many other areas of healthcare [5]. Cancer care imposes a substantial economic burden not only on society and healthcare systems but also on patients and their families and relatives [6,7]. Although there are major differences in healthcare costs for cancer among countries [8,9,10,11], additional patient out-of-pocket expenses are prevalent even in countries with universal healthcare systems or health insurance for all [12,13,14]. Health-insurance systems and health insurers are increasingly shifting costs of care to patients by raising deductibles and imposing copayments [15]. This creates significant discrepancies in the cost of cancer medication to the patient [16] because even if the cost of a given drug is fixed, it varies relative to household income and may influence persons with cancer in different ways [17,18]. 

Indeed, many studies have repeatedly shown the adverse financial consequences of the cost of cancer medications for patients and their families [7,19]. Most of these studies, however, were undertaken among cancer survivors and do not, to the best of our knowledge, focus on the financial burden on family members although this burden may remain and even continue to grow. Therefore, it is vital to take into account not only the patient’s out-of-pocket expenses and their economic consequences but also the ways in which patients’ entire families cope and live with the outcome. This study is novel as it focused specifically on the still relatively unexplored topic of surviving family members and the burden that they bear.

In cognizance of the financial burden on cancer patients and their family members and the negative financial consequences of pharmaceutical cancer care for patients and their families, the President’s Cancer Panel [20] acknowledged the need to address high cancer-drug prices as a national priority. In addition, the Cost of Care Task Force at the American Society of Clinical Oncology (ASCO) identified patient–physician cost communication as a critical component of high-quality care [21] and included high out-of-pocket expenses in a position statement that it released about the affordability of cancer drugs [20,22]. 

An important component of intervention aimed to alleviate the financial burden of cancer care is honest communication between oncologists and their patients [23]. Studies provide evidence that cost communication between patients and oncologists is associated with higher patient satisfaction and a lower financial burden due to smaller OOP payments and expenses [23,24]. However, even though patients report their wish to discuss the costs of their treatment [25] and patients, oncologists, and organizations agree that discussing out-of-pocket costs is important, such discussions are rare [26,27]. The main reason for this is that oncologists hesitate to discuss OOP costs with patients in the same way that they discuss the adverse effects of a treatment and appear reluctant to elaborate on and describe choices of care, believing that such a discussion sows uncertainty [1,28,29]. 

Either way, some aspects of the relation between cost communication and the burden of OOP payment on families after the patient’s death remain vague. Therefore, this study explored cost communication about the affordability of OOP medication at the end of life and the extent to which this communication on this topic is related to families’ financial burden after their loved ones’ death. 

## 2. Materials and Methods

### 2.1. Study Design

A cross-sectional survey was conducted in Israel among 491 relatives who had been primary caregivers (as reported in the medical records) of deceased cancer patients aged 23 and over, Jewish and Arab, who had been treated at the oncology departments of four medical centers. The names of 1000 consecutive patients who had died 3–6 months before the survey at these departments were retrieved from the centers’ medical records, as were the details of their relatives. A medical secretary contacted the relatives by telephone, briefly explained the survey to them, and asked them for their consent to be interviewed by telephone. Those who answered in the affirmative were interviewed by skilled interviewers. More than half (55.0%) of those who refused to be interviewed said that it is too difficult for them to speak about the last period of life of their loved one, 29.5% said that they had hardly had out-of-pocket expenses because their loved ones had deteriorated to death very quickly, and 15.5% said that they did not remember what their expenses had been.

Before the interview began, the interviewers read the consent document to the participants, who again assented to being interviewed. We decided to interview the patients’ relatives about all of our research questions, including those addressed to the patients, because the burden of answering a survey at the end of life was too high for terminal cancer patients to undertake and because some patients were too weak to speak or were unaware of the financial and administrative aspects of their care. Thus, the unity of the data collection was maintained. Since one of the main goals of the study was to study and track most accurately the expenses of cancer patients until their last day and due to the physical and mental difficulty of interrogating a patient in his or her last moments, the study made use of information collected by the patient’s close relatives.

A pre-test of 15 interviews preceded the interviews in order to determine the feasibility of the interview, the clarity of the questions, the interview guidance and face validity, response and non-response rates, and response quality. 

The data were analyzed by means of Stata 15 software and the level of significance was set at *p* < 0.05 (two-tailed). Indicators from descriptive statistics were used at first. Afterward, the relevant research variables were tested by means of linear and probability estimations.

### 2.2. Ethical Issues

The study was formally approved by the ethics committees of Tel HaShomer Medical Center, Emek Medical Center in Afula, Hadassah Medical Center, and Shaare Zedek Medical Center in Jerusalem. Each center’s Helsinki committee approved the study ethics, the procedure, and the survey questionnaire (4889-18-SMC, 0022-19-EMC, 0201-19-HMO, and 0285-18-SZMC, respectively). The participants were informed verbally that their answers would be kept secret for the purposes of the study and were required to declare their consent to this. All the participants were advised of the purpose of the study, the confidentiality of the information, and right to revoke their participation without prior justification.

### 2.3. Research Variables

The telephone interview questionnaire was designed to elicit information about the following:

**Out-of-pocket expenses:** The relatives were asked if they or the patients had paid out of pocket for medications up to six months before the patient’s death and, if so, how much. 

**Communication with oncologists:** The relatives were asked 2 questions: Did the oncologist who suggested a medication that is obtainable only by OOP payment take an interest in your financial ability to pay for out-of-pocket medication. Did he or she explain its advantages? And did he or she explain different treatment alternatives? Each question was rated on a 4-point scale from “to a large extent” to “not at all.”

**Economic burden** was measured with one question: To what extent did out-of-pocket expenditure create a financial burden for the patient and the whole family? The answers ranged from 1, “not at all”, to 4, “to a large extent.”

**Demographic and socioeconomic characteristics of the deceased patient:** The patient’s age at time of death, gender, education, religiosity, functional state, insurance coverage (private health insurance and/or long-term-care insurance, in addition to National Health Insurance), and the household’s ability to make ends meet.

## 3. Results

Among 491 relatives, 210 (42.77%) said that they and/or the patients had paid out of pocket for medications during the last six months of the patient’s life. The average age of the deceased patients was 68, 54% were women, and 50% had 13 years of education or more. About 43% were totally or almost totally incapacitated, 33% functioned with difficulty, and one-fourth were able to carry out activities of daily living. Fifty-five percent of patients’ households and relatives managed to make ends meet fairly easily or easily; all the others reported some or much difficulty. The average expenditure on medications in the last six months of the patient’s life was USD 5300 (Table 1). There were no socio-demographic or economic differences between those who had and had not incurred OOP expenses for medications.

**Figure 1:**
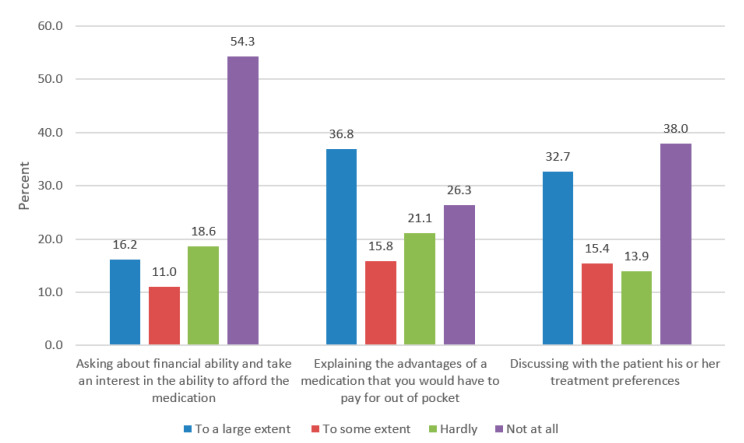
Explanation by physicians about out-of-pocket cost of medicinal care (%).

**Communication with oncologists:** According to the relatives, 73% of oncologists who suggested an OOP medication hardly asked or did not ask at all about financial ability and took little or no interest in ability to afford the medication, and 43% of them hardly explained or did not explain at all the advantages of an OOP medication that they recommended. Moreover, 52% of them hardly explained or did not explain any treatment alternatives (Figure 1). 

Table 2 focuses on variables that relate to oncologists’ communication about financial ability and about the advantages of OOP medication. The internal reliability (Cronbach’s α) of physicians’ asking about financial ability and explaining the advantages of an OOP medication (the first two questions in Figure 1) was found to be 0.6240; therefore, it was defined as a new variable: oncologists’ communication about an OOP medication and its advantages. The new variable is a continuous one based on a factor analysis of these two questions. Given that this variable is a continuous one, linear estimation using the OLS method was performed. All explanatory variables are exogenous, and a test to rule out multicollinearity was conducted [30]. 

The linear regression analysis revealed that the older the patients were, the less communication there was about an OOP medication and its advantages (B = −0.014, 95% CI = −0.028–0.001) and that oncologists communicated more with better-educated patients (B = 0.413, 95% CI = 0.052–0.773), more affluent patients, and those who had private health insurance (B = 0.669, 95% CI = 0.153–1.185) (Model 1). Oncologists made more reference to alternative treatment and the advantages of an OOP medication when communicating with financially better-off families. As for all the other explanatory variables, the direction of the explanation did not change but the extent of physicians’ interest and explanation was found to be higher in communication with male patients than with females (B = 0.297, 95% CI = −0.007–0.654) (Model 2).

**The burden of out-of-pocket payment for medication:** Almost one quarter (24.6%) of the relatives said that OOP payment for patients’ medication inflicted a very heavy financial burden on patients and their households, 30.7% reported a heavy burden, 24.6% a slight burden, and 21.1% no burden at all. The financial-burden variable is an ordinal one that ranged from 1, “not at all”, to 4, “to a large extent.” Therefore, it is more accurate statistically to use an ordinal method that brings into attention the meaning of values’ order instead of a multinomial method. We estimated the model both using an ordered logit regression and using an ordered probit regression, and the results were found to be consistent. Using an ordered logit regression analysis to estimate characteristics related to the financial burden, we found that the better educated a patient is, the lighter the financial burden (OR = 0.519, 95% CI = 0.279–0.967). The probability of a financial burden diminishes as the household’s ability to make ends meet improves: OR = 0.302, 95% CI = 0.101–0.899 among those who make ends meet with some difficulty; OR = 0.155, 95% CI = 0.049–0.492 among those who do so fairly easily; and OR = 0.062, 95% CI = 0.018–0.211 among those who do so easily. The probability of a financial burden is lower among those who have private health insurance (OR = 0.459, 95% CI = 0.193–1.093 for those with private health insurance) and among those inclined to religiosity (OR = 0.756, 95% CI = 0.581–0.983). In addition, the probability of a financial burden on the patient and her or his family declines in tandem with an increase in physicians’ interest in their financial ability and the extent of explanation that they provide as to the advantages and prospects of a medication that must be paid for out of pocket (OR = 0.750, 95% CI = 0.580–0.971) (Table 3).

## 4. Discussion

This study shows that patient–oncologist communication about the affordability of out-of-pocket purchase of medication is scarce. Although the patients were approaching the end of life (they died within half a year, and almost half of them were totally or almost totally incapacitated), only about one-fourth of the oncologists who suggested an OOP medication asked about financial ability and only about half explained the advantages of the medication in question. Thus, the current study corroborates Finlay and Casarett’s and Timmermans and Stivers’ [26,27] findings, which showed that a minority of patients considered themselves well informed about the costs of their cancer care prior to treatment and that most rarely spoke with their oncologist about the cost of care to themselves.

Various studies report a connection between oncologists’ suggestions of cancer drugs close to the end of patients’ lives and their difficulties in communicating with patients not only about cost but also about prognosis and other sensitive issues [31,32]. They trace this to concern about providing an incorrect prognosis, emotional discomfort, and lack of time to engage in difficult conversations [26]. 

The current study also showed that more than half of the families judged the financial burden of OOP spending on medications to be very heavy or heavy. Other studies have found a financial burden of cancer treatment among one-third to two-thirds of patients and their families [29,33,34,35,36]. The current study emphasizes the high degree of financial distress even among insured patients by reporting an onerous financial burden for medication in countries that offer generous entitlement to cost-free cancer medications under their national health insurance laws. 

Better education, stronger household ability to make ends meet, having private health insurance, and being inclined to religiosity diminish the probability of incurring a financial burden. Other studies have found similar socioeconomic characteristics reflected in lower household income and lower education level [37,38]. The lack of health-insurance coverage is also found to be one of the strongest correlates of medical financial hardship [7,39].

Controlling for patients characteristics, the probability of a financial burden caused by out-of-pocket spending on medication was significantly lower and physicians took more interest in the patient and the financial ability of the patient’s family and offered more explanation about the advantages and prospects of a medication that must be paid for out of pocket. Zafar and colleagues [23] also found an association between higher subjective financial distress and greater likelihood of wanting to discuss costs, and Meisenberg et al. [40] found that those who felt well informed were less likely to report that the cost of care had hurt them financially. Notably, however, while many people prefer to know about out-of-pocket costs before treatment, wish to discuss costs with their physician, and feel comfortable about doing so, some do not want costs to influence treatment irrespective of their degree of financial stress [41].

This study had several limitations. First, one may argue that relatives may not know enough about patients’ out-of-pocket spending. However, Israel is characterized by close ties and flows of support among family members and by families that bear major responsibility for relatives who meet with misfortune [42]. This, together with the fundamental belief in the paramount value of life, is twice as strong when cancer is involved. Therefore, we believe we can trust the knowledge of family members about their deceased relatives’ out-of-pocket spending. In general, the use of proxy respondents enhances research opportunities by allowing the inclusion of a broader range of situations and patients in research. Proxies have become particularly important in view of the increased use of outcome measures that do not rely on clinical tests [43]. The introduction of new treatments for Alzheimer’s disease, for example, was followed by a rapid increase in research on the costs and quality of life associated with the disease, much of which relies on proxy respondents [44]. In other studies, caregivers as key informants were asked about OOP expenditures in cases of patients who suffered from memory problems [45] and cancer [46]. 

Second, although we surveyed a consecutive sample of deceased cancer patients in their last six months of life, relatives who did not agree to be interviewed were excluded. This may have introduced selection and recall biases because families with high costs and high burdens are more likely to remember these costs and respond to survey requests. Additionally, the relatives interviewed may have not been present at discussions about costs, affordability, and treatment alternatives and thus did not realize that such talks had occurred. Furthermore, questions about financial burden are intrinsically subjective and may be interpreted differently by people of different means.

## 5. Conclusions

Concern about the cost and affordability of cancer drugs is widespread and well known among policymakers, service providers, insurers, oncologists, patients, and their relatives. This study, focusing on the last link in the chain—cancer patients’ relatives—revealed that discussion of and explaining the meaning of out-of-pocket payment for cancer drugs alleviates families’ experience of the financial burden associated with these expenses. 

Although the role of oncologists in this discussion is clear and gradually being accepted, their involvement in explaining out-of-pocket expenditure on drugs still leaves room for improvement. Explaining the complex interactions of cost and clinically meaningful outcomes is no easy task; therefore, oncologists need to be educated in skills that would enable them to communicate costs more openly and to consider the cost of a treatment when prescribing it. This approach has been endorsed by the American Society of Clinical Oncology, which recommends the development of a guideline statement on cancer costs [47] and defines financial counseling as an integral part of cancer care [33]. As we showed in this study, about 43% of patients received out-of-pocket drugs six months or less before their death. Therefore, as the ASCO suggests [48], oncologists should be taught not only how to discuss cost affordability but also how to discuss survival life expectancy when recommending out-of-pocket drugs. Oncologists play a central role not only in delivering high-quality medical treatment but also in coordinating different aspects of patient care such as managing depression, anxiety, and demoralization [49] as well as helping to contain the financial burden. This latter role pertains not only to patients but also to their family members, who will live with debts on account of OOP expenditure in the long term.

In addition, more research is needed to elucidate the influence of oncologists, the challenges they face in communicating about out-of-pocket costs of care, and what can be done to help them overcome their avoidance of the topic. There is also a need for better-oriented education, in all stages of physicians’ education, toward more overt communication in their practice and toward consideration of costs when they pursue a comprehensive approach to the care of cancer patients. 

## Figures and Tables

**Table 1:** Characteristics of deceased cancer patients (%).

Average Age (SD)	68.00 Years (13.17)
**Gender**	
Male	45.71
Female	54.29
**Education**	
1–8 years	9
9–12 years	41
13+ years	50
**State of functioning**	
Totally or almost totally incapacitated	42.86
Did things with difficulty	33.33
Was able to perform activities of daily living	23.81
**Insurance**	
Private health insurance	20.48
Long-term-care insurance	45.71
No private insurance (only National Health Insurance)	33.81
**Religiosity**	
Very religious	28.28
Somewhat	41.42
Secular	30.3
**Is your household able to make ends meet?**	
With great difficulty	12.95
With some difficulty	34.72
Fairly easily	26.94
Easily	25.39
**Terminal diagnosis**	
Digestive tract	20.1
Hepatobiliary	18.3
Hematology	14.7
NSCLC (pulmonary)	13.5
Breast	9.7
Prostate and urinary	8.6
GBM	7.5
Other	7.6
**Out-of-pocket expenditure on medications (SD) (USD)**	5300 (7787)

**Table 2:** Linear regression analysis to explain oncologists’ taking an interest in financial ability to pay for OOP medication and explaining its advantages.

	Model 1	Model 2
Male	0.175	0.297 *
	−0.179	−0.13
Age	−0.014 *	−0.017 **
	−0.007	−0.01
Education	0.413 *	0.215
	−0.182	−0.18
Make ends meet (reference: with great difficulty)		
With some difficulty	0.709 *	0.675 *
	−0.303	−0.29
Fairly easily	0.654 *	0.591 *
	−0.316	−0.26
Easily	0.738 *	0.663 *
	−0.332	−0.32
Insurance (reference: National Health Insurance only)		
Private health insurance	0.669 *	0.527 *
	−0.261	−0.25
Long-term-care insurance	0.135	0.092
	−0.204	−0.19
Religion	0.033	0.058
	−0.077	−0.07
Physicians explain different treatment alternatives		−0.294 ***
		−0.07
Constant	2.374 ***	3.440 ***
	−0.526	−0.57
Adj. R-squared	0.1368	0.2257
N	157	156

* *p* < 0.05, ** *p* < 0.01, *** *p* < 0.001. The numbers in parentheses are w standard errors.

**Table 3:** Ordered logit regression analysis to explain financial burden caused by out-of-pocket spending on medication (Explained variable: financial burden caused by out-of-pocket spending on medication: 1—none, 4—very large (odds ratio).

	Model 1
Male	1.162
	−0.356
Age	1.004
	−0.012
Education	0.519 *
	−0.165
Make ends meet (reference: with great difficulty)	
With some difficulty	0.302 *
	−0.168
Fairly easily	0.155 **
	−0.091
Easily	0.062 ***
	−0.039
Insurance (reference National Health Insurance only)	
Private health insurance	0.459 *
	−0.203
Long-term-care insurance	0.651
	−0.274
Religion	0.756 *
	−0.101
Oncologists’ taking an interest in financial ability to pay for OOP medication and explaining its advantages	0.750 *
	−0.099
Log-likelihood	−194.149
Pseudo R-squared	0.1809
N	157

* *p* < 0.05, ** *p* < 0.01, *** *p* < 0.001. The numbers in parentheses are standard errors.

## Data Availability

The data used in this study contains sensitive information about the study participants and their relatives did not provide consent for public data sharing. The current approvals by the ethics committees of Tel HaShomer Medical Center, Emek Medical Center in Afula, Hadassah Medical Center, and Shaare Zedek Medical Center in Jerusalem (reference numbers 4889-18-SMC, 0022-19-EMC, 0201-19-HMO, and 0285-18-SZMC, respectively) do not include data sharing.
